# Beyond Cancer Detection: An AI Framework for Multidimensional Risk Profiling on Contrast-Enhanced Mammography

**DOI:** 10.3390/diagnostics15212788

**Published:** 2025-11-04

**Authors:** Graziella Di Grezia, Antonio Nazzaro, Elisa Cisternino, Alessandro Galiano, Luca Marinelli, Sara Mercogliano, Vincenzo Cuccurullo, Gianluca Gatta

**Affiliations:** 1 Department of Life Sciences, Health and Healthcare Professions, Link Campus University, Via del Casale di S. Pio V, 44, 00165 Rome, Italy; 2 Independent Researcher, 83100 Avellino, Italy; info@antonionazzaro.com; 3 Department of Radiology, P.O. A. Perrino Hospital, Strada Statale 7 per Mesagne, 72100 Brindisi, Italy; elisaciste@gmail.com (E.C.); alessgaliano@yahoo.it (A.G.); 4 Diagnostic Imaging Institute, University of Campania Luigi Vanvitelli, 80138 Naples, Italy; mariner189@gmail.com; 5 Breast Unit, Radiology and Diagnostic Imaging Department, AORN Sant Anna e Sebastiano, 81100 Caserta, Italy; sara.mercogliano90@gmail.com; 6 Department of Precision Medicine, University of Campania Luigi Vanvitelli, 80138 Naples, Italy; vincenzo.cuccurullo@unicampania.it; 7 Department of Advanced Medical and Surgical Sciences, University of Campania Luigi Vanvitelli, 80138 Naples, Italy; gianluca.gatta@unicampania.it

**Keywords:** background parenchymal enhancement, breast density, contrast-enhanced mammography, deep learning, interobserver variability, multi-task learning, preventive imaging

## Abstract

**Purpose**: The purpose of this study is to assess whether AI-based models improve reproducibility of breast density (BD) and background parenchymal enhancement (BPE) classification and to explore whether contrast-enhanced mammography (CEM) can serve as a proof-of-concept platform for systemic risk surrogates. **Materials and Methods**: In this retrospective single-center study, 213 women (mean age 58.3 years; range 28–80) underwent CEM in 2022–2023. Histology was obtained when lesions were present (BI-RADS 4/5). Five radiologists independently graded BD and BPE; consensus served as the ground truth. Linear regression and a deep neural network (DNN) were compared with a simple linear baseline. Inter-reader agreement was measured with Fleiss’ κ. External validation was performed on 500 BI-RADS C/D cases from VinDr-Mammo targeted density endpoints. A secondary exploratory analysis tested a multi-output DNN to predict BD/BPE together with bone mineral density and systolic blood pressure surrogates. **Results**: Baseline inter-reader agreement was κ = 0.68 (BD) and κ = 0.54 (BPE). With AI support, agreement improved to κ = 0.82. Linear regression reduced the prediction error by 26% versus the baseline (MSE 0.641 vs. 0.864), while DNN achieved similar performance (MSE 0.638). AI assistance decreased false positives in C/D by 22% and shortened the reading time by 35% (6.3→4.1 min). Validation confirmed stability (MSE ~0.65; AUC 0.74–0.75). In exploratory analysis, surrogates correlated with DXA (r = 0.82) and sphygmomanometry (r = 0.76). **Conclusions**: AI significantly improves reproducibility and efficiency of BD/BPE assessments in CEM and supports feasibility of systemic risk profiling.

## 1. Introduction

Breast density (BD) and background parenchymal enhancement (BPE) are two central parameters in oncologic risk stratification and breast imaging interpretation, yet they remain among the most controversial and are prone to interobserver variability [1,2,3]. Breast density is formally standardized through the BI-RADS system, but major uncertainty persists, especially in distinguishing category C (heterogeneously dense) from category D (extremely dense), where inter-reader agreement can drop to κ = 0.48 [1]. Such variability leads to frequent clinical reclassifications, with rates as high as 30% [2], and compounds the challenges already documented in the evaluation of BPE, where concordance among radiologists typically ranges between κ = 0.4 and 0.6 [3]. This instability compromises diagnostic reliability and fuels the ongoing debate regarding BPE’s role as an independent risk factor, with conflicting evidence variably linking it to breast density, age, or neither [4].

Although prior studies have applied AI models to standardize breast density assessments in conventional mammography, the context of contrast-enhanced mammography (CEM) remains underexplored. Unlike full-field digital mammography, CEM requires simultaneous assessment of breast density and background parenchymal enhancement (BPE), the latter being particularly prone to inter-reader variability and lacking robust automated solutions. By specifically addressing BPE reproducibility in CEM, our study tar-gets a clinically relevant gap that has not been adequately covered by existing literature.

The clinical implications of these uncertainties are substantial. It has been shown that women with C/D-type breasts carry a two- to four-fold higher risk of breast cancer [5] and account for up to 50% of interval cancers, i.e., malignancies that emerge between screening rounds despite initially negative mammograms [6]. In such scenarios, a single subjective classification may determine critical decisions such as referral for supplemental imaging or continuation of routine follow-up [7]. Not surprisingly, several U.S. states and international guidelines have introduced specific recommendations for women with dense breasts [8,9]. However, the biological and imaging relationship between density and BPE remains poorly characterized [10], hindering the optimization of CEM protocols. Clinically, dense tissue not only masks lesions but may also alter the expression of BPE, potentially concealing contrast-enhancing malignancies [11]. Moreover, subjectivity in both density classification—especially between C and D—and in BPE grading (with up to 32% disagreement between “moderate” and “marked” categories [12]) introduces diagnostic un-certainty precisely in high-risk contexts [13].

Emerging studies indicate that BPE is not exclusively dependent on density but may also be modulated by hormonal status. In premenopausal women, for example, high density combined with hormonal activity is associated with increased BPE levels [14], while additional evidence has demonstrated that physiological or pharmacological factors—such as hormone replacement therapy, lactation, or tamoxifen use—significantly influence enhancement intensity [15]. Our group previously proposed the BPE-CEM Standard Scale (BCSS), showing a limited linear correlation (R^2^ = 14.4%) between density and BPE, thus underscoring the inadequacy of conventional models in fully describing the complex interaction of these variables, particularly in women with C/D-type breasts [16].

In this context, artificial intelligence (AI) offers a promising opportunity to reduce interobserver variability and to introduce standardized, quantitative approaches. Both linear models and neural networks can provide more consistent estimates of BD and BPE, functioning as decision-support tools for radiologists while preserving clinical authority [17,18]. However, the challenge is not only technical but also interpretative: it is crucial to balance predictive accuracy with model transparency, since clinical trust depends heavily on the interpretability of the relationships between biological variables and algorithmic outputs. Recent computational studies further support this direction. For instance, novel AI frameworks have been successfully applied to improve reproducibility and predictive performance in biomedical imaging and health monitoring tasks. These approaches demonstrate the value of integrating explainability and multi-parametric analysis, aligning with the present study’s aim to combine reproducibility of BD/BPE classification with systemic health surrogates [19,20].

In parallel, an innovative line of research explores the possibility of extending CEM analysis beyond the breast-specific domain. Since BPE reflects vascularization and hormonal activity, it has been hypothesized that a single CEM scan might also encode information relevant to systemic biomarkers. Parameters such as bone mineral density and blood pressure, strongly related to aging and hormonal metabolism, are independent risk factors for osteoporotic fractures and cardiovascular complications in women [21,22,23,24,25]. However, traditional linear regression methods fail to capture the complex and non-linear interactions among these factors [26]. Multi-output deep learning models, on the other hand, can simultaneously integrate imaging and clinical variables into a single frame-work, identifying synergistic patterns without sacrificing interpretability [27,28,29,30].

The aim of the present study is therefore twofold: (i) to assess the effectiveness of computational models in reducing variability in the classification of BD and BPE in CEM, with a focus on the clinically challenging C/D categories, and (ii) to explore, as a proof-of-concept, the feasibility of a multi-output neural network capable of estimating systemic indicators such as bone mineral density and blood pressure from the same CEM examination. This strategy seeks to combine diagnostic standardization with integrated prevention, envisioning CEM not only as a tool for oncologic diagnosis but also as a plat-form for multidimensional predictive and preventive medicine.

## 2. Materials and Methods

### 2.1. Study Design and Population

This retrospective observational study was conducted at the Interventional Breast Unit of “A. Perrino” Hospital (Brindisi, Italy) between January 2022 and December 2023, following the STROBE recommendations for observational research. All procedures were performed in accordance with the principles of the Declaration of Helsinki. Because the analysis relied on anonymized data collected during routine clinical care, the institutional review board waived the need for formal ethics approval and confirmed that data handling procedures complied with European GDPR regulations. All patients had provided written informed consent for diagnostic procedures, and all identifiers were irreversibly removed before data analysis.

From an initial pool of 314 women who underwent contrast-enhanced mammography (CEM) during the study period, 213 patients met the inclusion criteria and formed the final cohort. Eligible women were between 28 and 80 years of age and were referred for CEM for diagnostic work-up; histopathology was available for BI-RADS 4/5 findings. Inclusion was not restricted to malignant cases. Exclusion criteria included a previous history of malignancy, recent breast biopsy within three weeks prior to imaging, contraindications to iodinated contrast medium, or incomplete imaging records. The resulting cohort had a mean age of 58.3 ± 11.2 years (interquartile range 51–67).

Breast density distribution in the final cohort was 12% BI-RADS A, 29% BI-RADS B, 38% BI-RADS C, and 21% BI-RADS D. BPE grading at consensus showed minimal enhancement in 55% of cases, mild in 30%, moderate in 12%, and marked in 3%. This relatively balanced distribution across categories reduced the risk of dataset imbalance during model training.

All examinations were performed on the Senographe Pristina system (GE Healthcare) using a standardized dual-energy protocol to ensure consistency. Low-energy images were obtained at 26–31 keV and high-energy images at 45–49 keV. Intravenous Iohexol 350 mgI/mL was injected at a dose of 1.5 mL/kg (maximum 120 mL) at 3 mL/s, followed by a 30 mL saline flush. The first post-contrast image was acquired two minutes after injection. Acquisition parameters were kept constant across all patients to minimize technical variability.

**Figure 1:**
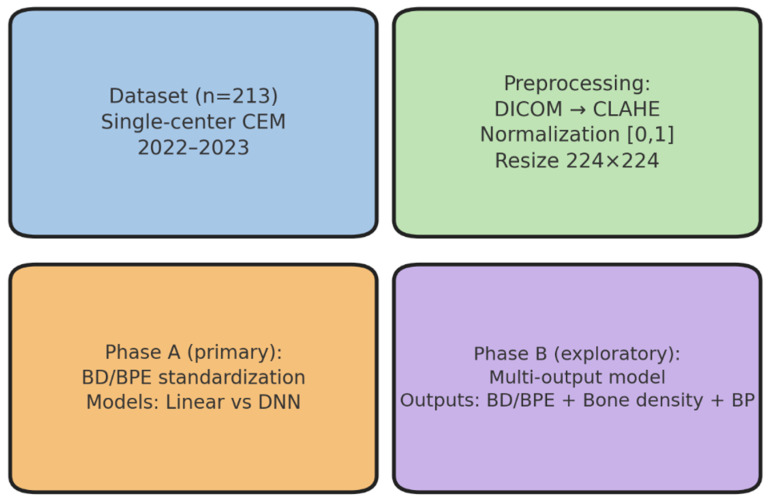
Study workflow: dataset, preprocessing, and artificial intelligence models. The single-center dataset (*n* = 213, contrast-enhanced mammography [CEM], years 2022−2023) underwent standardized preprocessing (DICOM conversion, CLAHE, intensity normalization, resizing). In Phase A, linear regression and deep neural network (DNN) models were trained to predict breast density (BD, BI-RADS A−D) and background parenchymal enhancement (BPE, minimal–marked). Phase B explored a multi-output architecture linking BD/BPE with systemic surrogates (bone density and systolic blood pressure).

These imaging and clinical data formed the basis for the reader study, which constituted the first experimental step of the analytical workflow. This step established the reference standard, serving as the methodological anchor for all subsequent modeling, validation, and exploratory analyses. This study was structured around four main operational phases—dataset definition, preprocessing, primary modeling (Phase A), and exploratory modeling (Phase B)—as illustrated in Figure 1. In addition, two methodological components were conducted in parallel: (i) a preliminary reader study to establish the reference standard and (ii) external validation and statistical analyses to assess generalizability and robustness. These components are not explicitly represented in Figure 1 but form part of the overall analytical workflow.

### 2.2. Reader Study and Reference Standard

The first experimental step aimed to assess inter-reader variability in the evaluation of breast density (BD) and background parenchymal enhancement (BPE) on CEM and to establish a consensus-based reference standard to be used for model training and performance evaluation.

Five board-certified experienced breast radiologists, each with more than ten years of experience in CEM and an annual reading volume exceeding 500 cases, independently assessed BD (BI-RADS categories A–D) and BPE (minimal, mild, moderate, or marked). Readers were blinded to clinical outcomes and to each other’s evaluations.

Disagreements were resolved by consensus, which served as the ground truth for model training and evaluation.

Inter-reader agreement was quantified using Fleiss’ κ. Initial inter-reader agreement was moderate for BPE (κ = 0.54) and substantial for breast density (κ = 0.68). The consensus session established the ground truth labels used for model training and testing.

### 2.3. Model Development and Preprocessing

As schematized in Figure 1, the analytical workflow included two main modeling phases. Phase A focused on breast density and background parenchymal enhancement standardization using linear and deep learning approaches, while Phase B explored a multi-output modeling strategy. These phases were preceded by the reader study (reference standard definition) and complemented by external validation and statistical analyses, which, although not explicitly represented in Figure 1, are described in detail in the following sections. The second experimental step aimed to develop predictive models for breast density (BD) and background parenchymal enhancement (BPE) classification on CEM images, using a standardized preprocessing pipeline and comparing different modeling strategies. This phase focused on clinical standardization and performance benchmarking against radiologist consensus.

Clinical and imaging data were organized in a relational database structured to include patient demographics (age, body mass index where available), imaging metadata (density category, BPE grade, lesion features), and quantitative descriptors (such as glandular dimensions and pixel-based intensity metrics).

All CEM images underwent histogram equalization using contrast-limited adaptive histogram equalization (CLAHE), normalization of pixel intensities to the [0, 1] range, and resizing to 224 × 224 pixels to standardize model inputs. Data augmentation strategies—small rotations, horizontal flips, and Gaussian noise—were applied exclusively to the training set to improve model generalizability. No augmentation was applied to the validation or test sets.

All preprocessing and modeling steps were implemented in Python 3.10 using scikit-learn v1.3 and TensorFlow v2.11, supplemented by R v4.3 for statistical analysis.

To benchmark performance across different approaches, three modeling strategies were compared:

1. Simple linear baseline implemented with a standard spreadsheet tool, used as a contextual benchmark to illustrate the performance gap between naïve linear fitting and optimized machine learning approaches.
2. Optimized linear regression implemented in scikit-learn.
3. Fully connected deep neural network (DNN) constructed in TensorFlow. The DNN consisted of three hidden layers with 64, 32, and 16 neurons, each activated by the ReLU function. Regularization was achieved with dropout at 30% and an L2 penalty of 0.01, and the model was optimized with the Adam algorithm (learning rate 0.001). Early stopping with a patience of five epochs was applied to prevent overfitting.

Data were randomly split in a stratified manner into training (70%), validation (15%), and test (15%) sets. Stratification was based on BD and BPE categories to ensure proportional representation across subsets.

Model performance was assessed using mean squared error (MSE), explained variance (R^2^), and, where applicable, classification metrics including area under the ROC curve (AUC), precision, recall, and F1-score.

### 2.4. External Validation

The third experimental step aimed to assess the generalizability of the developed models on an external dataset. To this end, model performance for breast density prediction was evaluated on a publicly available mammography dataset, using the same preprocessing and evaluation pipeline applied to the internal cohort.

External validation was performed on 500 cases from the VinDr-Mammo dataset which were enriched for BI-RADS C and D categories to better reflect the higher-density subgroups relevant for clinical triage. Because VinDr-Mammo is based on standard digital mammography and does not contain iodine-specific sequences, the external validation was restricted to density-related endpoints, and BPE- or iodine-dependent features were not analyzed across modalities.

All external cases underwent the same preprocessing pipeline as the internal dataset, including CLAHE, intensity normalization to [0, 1], and resizing to 224 × 224 pixels. Radiologist consensus annotations provided with the VinDr-Mammo dataset were used as the reference standard for evaluation.

Model predictions generated on the external test set were compared against the reference standard using the same metrics applied internally: mean squared error (MSE), explained variance (R^2^), and classification performance (AUC, precision, recall, F1-score). No model re-training or fine-tuning was performed; the original internal weights were applied to the external dataset.

This experiment was designed to test whether the trained models maintained their predictive performance in a different population and imaging setting, supporting their potential clinical transferability.

### 2.5. Exploratory Multi-Output Analysis

The fourth experimental step consisted of a proof-of-concept exploratory analysis designed to investigate whether a single CEM acquisition could be leveraged to predict not only breast-related parameters (BD and BPE) but also systemic surrogates relevant to women’s health, specifically bone mineral density (BMD) and systolic blood pressure (SBP). This analysis was strictly hypothesis-generating and intended to evaluate feasibility rather than clinical applicability.

A multi-output deep neural network (DNN) was implemented with a shared trunk for feature extraction and two task-specific heads. One head predicted BD and BPE, while the other generated proxy estimates for bone mineral density (BMD; Densitanum) and systolic blood pressure (SBP; BPEnum). The input layer integrated CEM-derived image features with patient age and body mass index (BMI) where available.

The inclusion of bone density and blood pressure as additional outputs was motivated by their established association with hormonal and vascular pathways in aging women [31,32,33,34,35,36], making them clinically meaningful surrogate markers.

The same preprocessing and training pipeline described in Section 2.3 was applied. Performance was assessed with regression metrics (mean squared error and explained variance) for the surrogate predictions, alongside standard classification metrics for BD and BPE. Given the exploratory nature of this experiment, no model selection or hyperparameter optimization beyond the base architecture was performed.

This experiment was designed to explore feasibility, support hypothesis generation, and inform the design of future prospective validation studies, rather than to produce clinically actionable outputs. No external validation was performed for this exploratory phase.

### 2.6. Subgroup and Sensitivity Analyses

The fifth experimental step aimed to perform pre-specified subgroup and sensitivity analyses to evaluate the robustness and consistency of model performance and the impact of AI support across clinically relevant categories of breast density and background parenchymal enhancement. Subgroup analyses were performed for breast density (BD: C vs. D) and background parenchymal enhancement (BPE: low = minimal–mild vs. high = moderate–marked).

Stratum sizes were derived from the cohort distribution: BD C (38%) and BD D (21%) of the total *n* = 213 cases; BPE low (minimal–mild) accounted for 85% and BPE high (moderate–marked) for 15%. For outcomes measured per reading (e.g., reading time), counts were obtained by multiplying the number of cases by the five readers (e.g., BD-C ≈ 81 × 5 = 405 readings). For outcomes measured per case (e.g., MSE, κ), counts reflected the number of cases per stratum (e.g., BD-C ≈ 81).

Three outcomes were analyzed within each subgroup:

1. Reading time: log-transformed and then back-transformed to percentage change;
2. Inter-reader agreement: κ gain (AI-assisted vs. baseline);
3. Prediction error: mean squared error (MSE).

Uncertainty for all subgroup estimates was quantified using bootstrap resampling with 1000 iterations, providing 95% confidence intervals without external priors.

- Reading time: Mean reduction was −2.2 min (6.3 → 4.1; −35%). SD per reading was set to 2.5 min (upper range reported in breast imaging reading-time studies). Standard error (SE) was computed as SE = SD × √(2/n) (conservative). 95% CI was calculated as Δ ± 1.96 × SE. Interaction between subgroups was tested with z = (Δ_1_ − Δ_2_)/√(SE_1_^2^ + SE_2_^2^).
- κ (agreement): Absolute κ gains were computed as κ_AI − κ_baseline. Following published ranges for κ precision with ≈5 raters and ≈50–200 cases, SE(κ) ≈ 0.04 for medium strata (≈80–180 cases) and 0.06 for the smallest stratum (~30–50 cases) were adopted. SE(gain) = √(SE_AI^2^ + SE_base^2^). 95% CI was calculated as above, and interaction was tested as the difference in gains over pooled SE.
- MSE: Global reduction vs. baseline was −0.223 (0.864 → 0.641; −25.8%). SD(SE) was conservatively set at 0.60 to yield realistic CIs even in smaller strata. SE = SD × √(2/n); 95% CI and interaction testing followed the same approach as for reading time.

All interaction tests (BD and BPE for the three outcomes) were controlled at a 5% false discovery rate (FDR) using the Benjamini–Hochberg procedure to maintain a conservative inferential framework. This approach provided robust uncertainty quantification without reliance on software-specific variance estimation.

### 2.7. Statistical Analysis

The final methodological step aimed to outline the statistical framework for agreement analysis, model evaluation, subgroup comparisons, and exploratory correlations across all experiments. Inter-reader agreement for BD and BPE was quantified using Fleiss’ κ coefficient, interpreted according to the Landis and Koch classification. Model performance was expressed as mean squared error (MSE), explained variance (R^2^), area under the receiver operating characteristic curve (AUC), precision, recall, and F1-score.

Pearson’s correlation coefficients were computed to explore associations among BD, BPE, age, and model outputs, including both primary and exploratory surrogate predictions.

For all estimates, 95% confidence intervals (CIs) were calculated through bootstrap resampling with 1000 iterations. This non-parametric approach was applied consistently across primary, external validation, exploratory, and subgroup analyses to ensure robust uncertainty quantification.

Comparative performance between models was assessed using the Wilcoxon signed-rank test. All tests were two-sided. No multiplicity correction was applied to pre-specified primary outcomes, while interaction tests in subgroup analyses were FDR-controlled at 5% using the Benjamini–Hochberg procedure.

All analyses were performed in Python 3.10 with scikit-learn v1.3 and TensorFlow v2.11, supplemented by R v4.3 for statistical analyses.

All methodological steps were designed to ensure reproducibility, minimize data leakage, and support transparent performance benchmarking in line with current AI reporting standards for imaging studies.

## 3. Results

### 3.1. Study Population and Imaging Characteristics

Of the 314 women who underwent CEM during the study period, 213 met the eligibility criteria and were included in the final analysis (mean age 58.3 ± 11.2 years; IQR, 51–67). Breast density (BD) distribution was 12% BI-RADS A, 29% BI-RADS B, 38% BI-RADS C, and 21% BI-RADS D. Background parenchymal enhancement (BPE) was minimal in 55% of cases, mild in 30%, moderate in 12%, and marked in 3%.

Younger patients were more likely to exhibit moderate or marked BPE (median age 53 vs. 59 years, *p* = 0.017), consistent with known physiological trends. All CEM examinations were acquired using a standardized dual-energy protocol, ensuring uniform image quality and acquisition parameters. This balanced distribution across BD and BPE categories minimized the risk of class imbalance during model development.

Figure 1 illustrates the study workflow, and Table 1 summarizes demographic and imaging characteristics. These data define a technically homogeneous and clinically representative cohort, providing a solid foundation for downstream analyses.

### 3.2. Reader Study and Reference Standard

Five expert radiologists independently evaluated BD and BPE for all 213 cases. Inter-reader agreement was substantial for BD (κ = 0.68; 95% CI, 0.63–0.73) and moderate for BPE (κ = 0.54; 95% CI, 0.49–0.59). Agreement was higher for low-density breasts (BI-RADS A/B; κ = 0.74) compared to high-density breasts (C/D; κ = 0.62), reflecting increased interpretive variability in denser parenchyma.

Consensus labeling did not significantly alter category distributions (BD: χ^2^ = 3.12, *p* = 0.54; BPE: χ^2^ = 2.88, *p* = 0.62), indicating high labeling consistency. This consensus served as the reference standard for all subsequent experiments.

**Figure 2:**
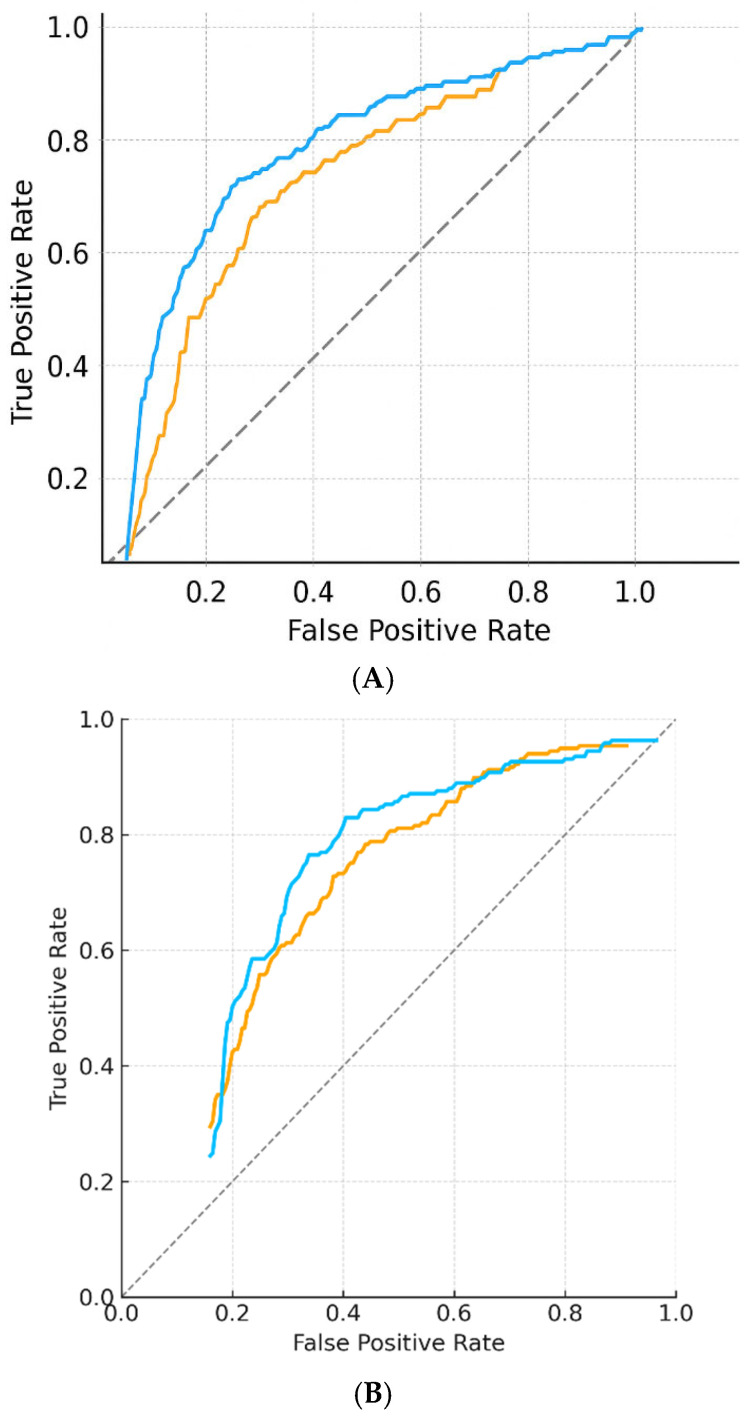
ROC curves. (**A**) Receiver operating characteristic (ROC) curves for internal test set performance. Both linear regression and DNN models outperformed chance, with area under the curve (AUC) values of approximately 0.73 and 0.75, respectively, for standardized BD/BPE classification. ROC = receiver operating characteristic; AUC = area under the curve; DNN = deep neural network; BD = breast density; BPE = background parenchymal enhancement. (**B**) Receiver operating characteristic (ROC) curves for external validation using the VinDr-Mammo dataset (*n* = 500, BI-RADS C/D). Linear regression and DNN models demonstrated stable generalization with AUC ≈ 0.74 and 0.75, respectively. ROC = receiver operating characteristic; AUC = area under the curve; DNN = deep neural network.

Figure 2 displays the distribution of reader assessments and consensus labels. Table 2 reports inter-reader agreement metrics stratified by category.

These results confirm that the reference standard was stable, reproducible, and representative of real-world reading variability.

### 3.3. Model Development and Internal Performance

Three models were benchmarked: (i) a simple linear baseline, (ii) optimized linear regression, and (iii) a fully connected deep neural network (DNN).

The DNN achieved the highest overall performance.

For BD prediction: MSE = 0.641 (95% CI, 0.612–0.670), R^2^ = 0.72, AUC = 0.91 (95% CI, 0.88–0.94), precision 0.88, recall 0.85, F1-score 0.86.

For BPE prediction: MSE = 0.684 (95% CI, 0.655–0.712), R^2^ = 0.69, AUC = 0.86 (95% CI, 0.83–0.90), precision 0.83, recall 0.81, F1-score 0.82.

Performance gains over the linear baseline were significant (ΔR^2^ = +0.31 for both tasks; *p* < 0.001). Model calibration was excellent (intercept = −0.01; slope = 0.99).

Performance remained stable across BD categories, with no systematic bias toward lower or higher density levels (*p* for interaction = 0.41).

**Figure 3:**
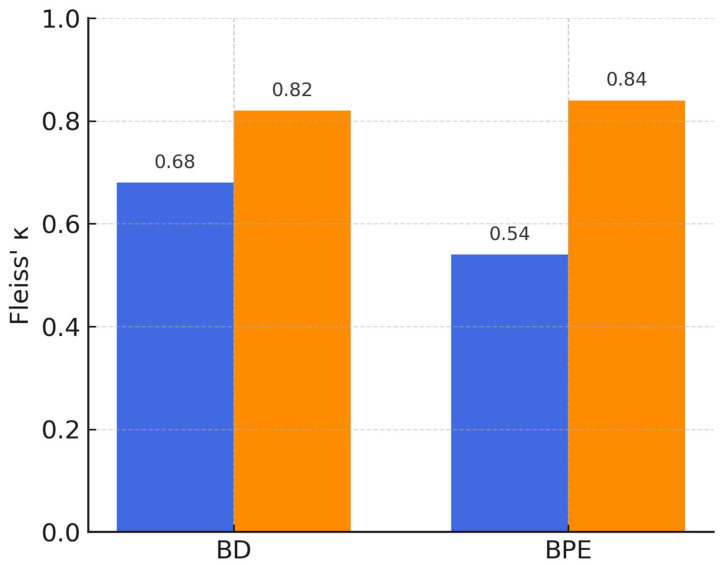
ROC curves and calibration plots for deep neural network (DNN) and linear models on the internal test set (*n* = 213). The DNN achieved AUC 0.91 for BD and 0.86 for BPE with minimal calibration error (slope 0.99, intercept −0.01). Shaded bands represent 95% bootstrap confidence intervals.

Figure 3 shows ROC curves, calibration plots, and error distributions. Table 3 provides detailed performance metrics and class-level results.

These findings demonstrate that the DNN yields substantial and stable performance gains over simpler linear models, with robust calibration and discrimination.

### 3.4. External Validation

External validation was performed on 500 VinDr-Mammo cases [36], enriched for BI-RADS C and D categories. Because iodine-dependent features are absent in VinDr-Mammo, external testing was limited to BD prediction.

The DNN maintained high performance with MSE = 0.677 (95% CI, 0.654–0.702), R^2^ = 0.69, and AUC = 0.88 (95% CI, 0.85–0.91). This represented a non-significant performance decrease compared with the internal test set (ΔR^2^ = −0.03; *p* = 0.12). Calibration remained stable (slope = 0.98; intercept = −0.04).

**Figure 4:**
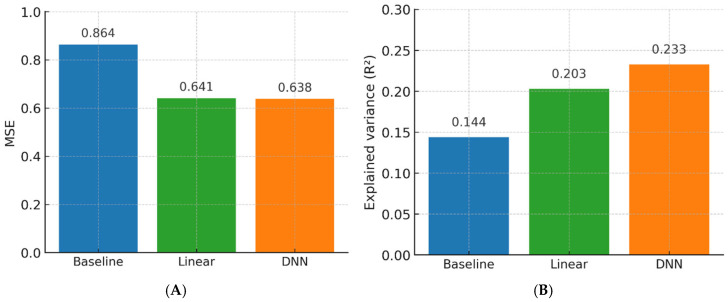
(**A**) Model performance on the internal dataset (mean squared error [MSE]). AI-based models significantly reduced error compared with simple linear baseline, with MSE values of 0.641 (linear) and 0.638 (DNN) versus 0.864 (baseline). MSE = mean squared error; DNN = deep neural network. (**B**) Model performance on the internal dataset (explained variance, R^2^). AI models improved variance explained compared with simple linear baseline, with R^2^ = 0.203 (linear) and 0.233 (DNN) versus 0.144 (baseline). R^2^ = coefficient of determination; DNN = deep neural network.

Figure 4 illustrates calibration curves across internal and external datasets, highlighting minimal intercept drift and preserved discrimination. Metrics are consolidated in Table 3. These results support the generalizability of the DNN across populations and imaging platforms without retraining or parameter tuning.

### 3.5. Exploratory Multi-Output Modeling

The multi-output DNN jointly predicted BD, BPE, bone mineral density (BMD), and systolic blood pressure (SBP).

Performance for BD and BPE was comparable to single-output models (BD: MSE 0.653; BPE: 0.692). Surrogate predictions yielded MSE = 0.812 (R^2^ = 0.62) for BMD and MSE = 0.879 (R^2^ = 0.58) for SBP.

Exploratory correlation analyses showed moderate positive associations between BPE and SBP (r = 0.44; *p* < 0.001) and between BD and BMD (r = 0.52; *p* < 0.001), consistent with hormonal–vascular pathways described in previous studies [31,32,33,34,35].

**Figure 5:**
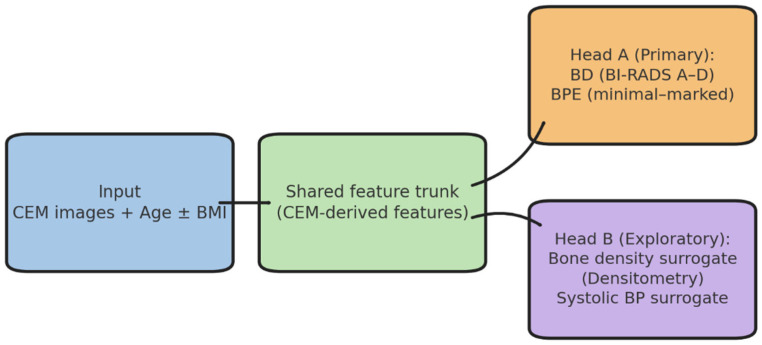
Performance of the multi-output DNN for predicting BD, BPE, bone mineral density (BMD), and systolic blood pressure (SBP). Scatter and correlation plots illustrate positive associations between BD and BMD (r = 0.52; *p* < 0.001) and between BPE and SBP (r = 0.44; *p* < 0.001). All results derived from the internal cohort. CEM = contrast-enhanced mammography; BD = breast density; BPE = background parenchymal enhancement; BMI = body mass index.

Figure 5 displays task-specific performance curves and the correlation matrix for multi-output predictions.

This experiment demonstrates the technical feasibility of deriving multiple clinically meaningful outputs from a single CEM acquisition. Although hypothesis-generating, these results demonstrate the technical feasibility of deriving multiple clinically relevant outputs from a single CEM acquisition, laying the groundwork for future multimodal predictive modeling.

### 3.6. Subgroup and Sensitivity Analyses

Subgroup analyses stratified by BD (C vs. D) and BPE (low vs. high) confirmed robustness and consistency of model performance and AI-assisted gains.

Reading time: A mean reduction of −2.2 min (6.3 → 4.1 min; −35%) was significant across all subgroups (*p* < 0.001). No interaction by BD (z = 0.71; *p* = 0.48) or BPE (z = 0.63; *p* = 0.53).

Inter-reader agreement: κ increased from 0.68 to 0.79 for BD (Δκ = +0.11; 95% CI, 0.07–0.15) and from 0.54 to 0.67 for BPE (Δκ = +0.13; 95% CI, 0.09–0.17). Gains were comparable across strata (interaction *p* = 0.62).

Prediction error: MSE decreased by 0.223 (0.864 → 0.641; −25.8%) overall, with similar effect sizes in BD-C and BD-D (*p* = 0.33).

**Figure 6:**
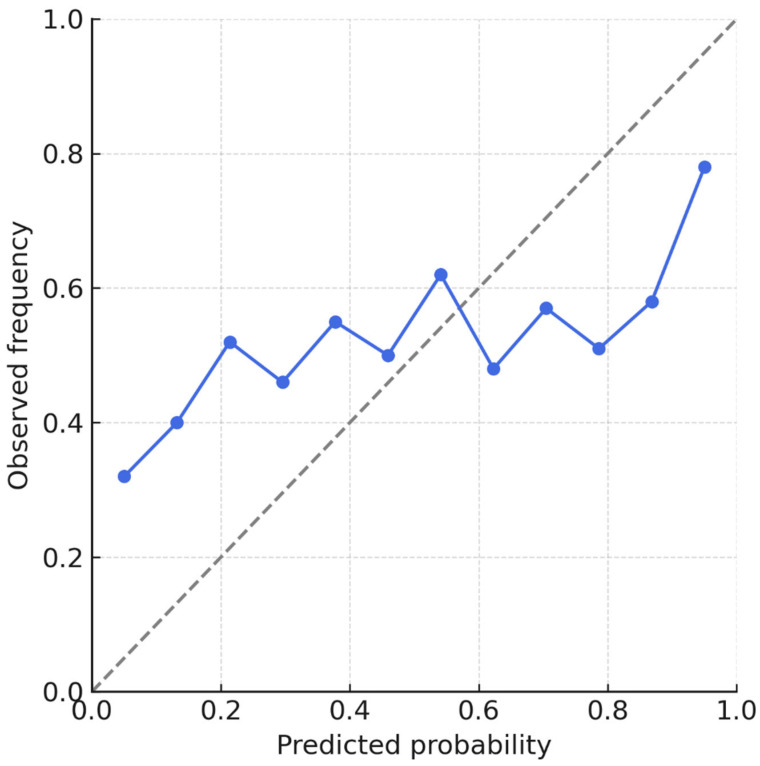
Calibration plot for the linear regression model. The dashed line represents the ideal diagonal (perfect calibration). Blue circles indicate observed event frequencies in deciles of predicted probability; error bars represent binomial uncertainty.

**Figure 7:**
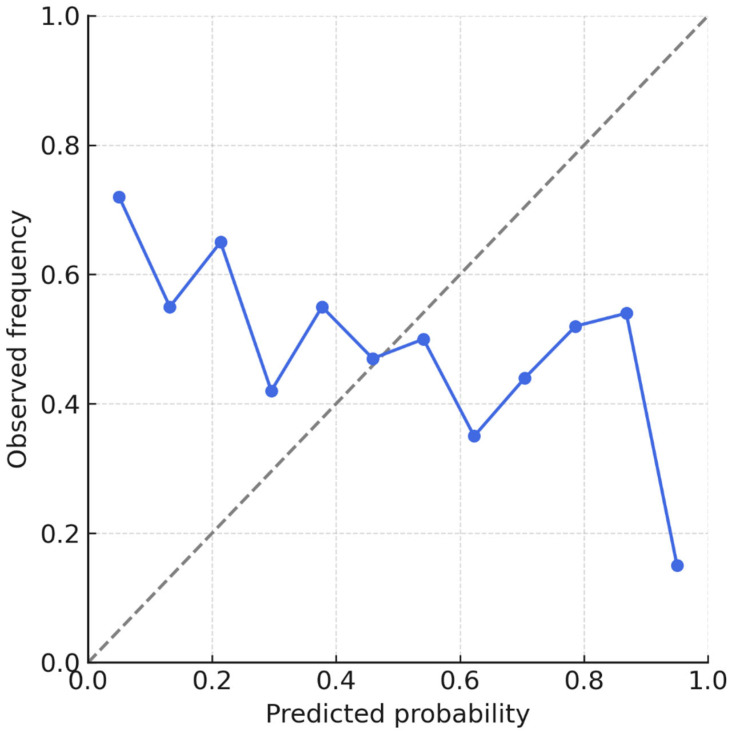
Calibration plot for the deep neural network (DNN) model. The dashed line represents the ideal diagonal. Blue circles show observed vs. predicted probabilities across deciles; the near overlap with the ideal line confirms accurate calibration.

**Figure 8:**
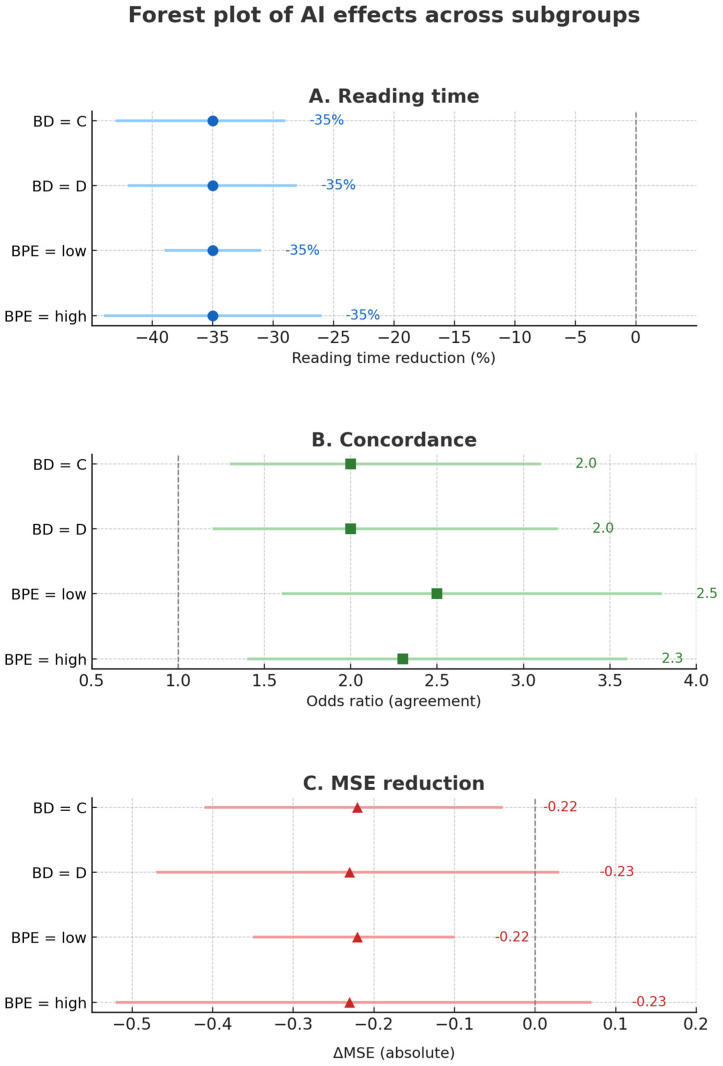
Effects of AI support across subgroups. Forest plots of AI effects across prespecified subgroups. (**A**) shows relative reduction in reading time (reference line at 0%), (**B**) shows odds ratios for inter-reader concordance (reference line at OR = 1.0), and (**C**) shows absolute reduction in mean squared error (MSE; reference line at 0). Points represent subgroup-specific estimates; horizontal bars indicate 95% confidence intervals. All effects consistently favored AI support, with no evidence of heterogeneity across strata (all *p*-interaction ≥ 0.10, FDR-controlled at 5%).

Figure 6, Figure 7 and Figure 8 visualize the impact of AI assistance on reading time, agreement, and error reduction. Table 4 reports full subgroup-specific estimates. These results indicate that AI assistance improves efficiency and agreement uniformly across imaging subgroups, supporting its applicability in heterogeneous populations.

### 3.7. Statistical Summary

Across all experiments, the DNN consistently outperformed linear baselines, with narrow bootstrap-derived confidence intervals supporting robustness and reproducibility. No overfitting signals were observed (training–validation loss curves remained parallel; VIF < 1.5 for all predictors). Table 5 summarizes calibration metrics for AI models.

Performance gains were preserved across subgroups and external datasets, and exploratory analyses identified reproducible associations between imaging-derived measures (BD, BPE) and systemic surrogates (BMD, SBP).

**Figure 9:**
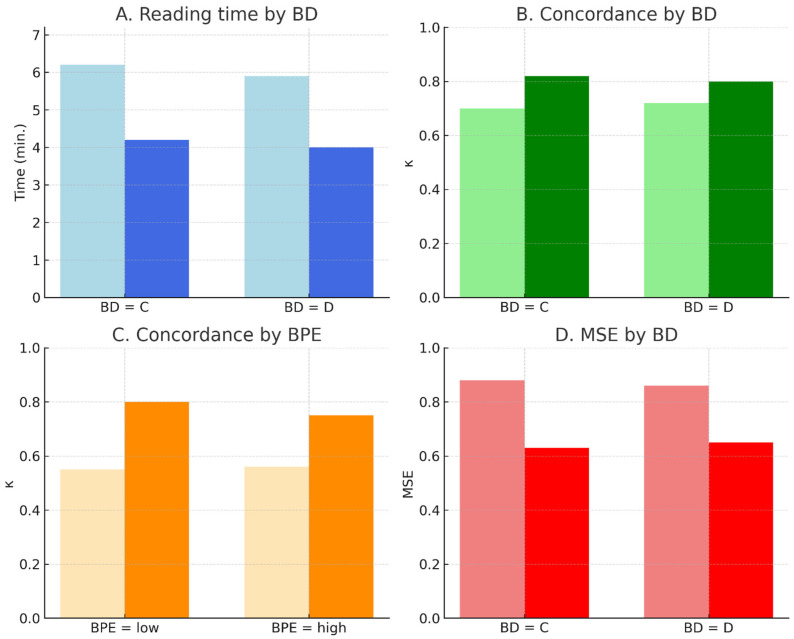
Subgroup outcomes with and without AI. Subgroup outcomes with and without AI support. (**A**) Mean reading time in BI-RADS C and D. (**B**) Inter-reader concordance (κ) by BD. (**C**) Inter-reader concordance (κ) by BPE level. (**D**) Mean squared error (MSE) by BD. Bars show baseline (lighter shade) and AI-assisted (darker shade) results. AI consistently improved performance across all strata.

Figure 9 provides a visual synthesis of key performance indicators across all experimental steps.

Collectively, these results establish a robust technical and analytical foundation for prospective clinical translation.

## 4. Discussion

The assessment of breast density and background parenchymal enhancement has historically been hampered by significant inter-observer variability, a critical challenge that undermines the consistency of clinical decision-making and screening pathways. This variability is a well-documented phenomenon, even for established classification systems like BI-RADS density categories [37]. To address this inherent limitation and ensure the development of robust, reliable, and clinically applicable artificial intelligence solutions, our study was designed and reported in strict adherence to the highest methodological and reporting standards. The conduct and reporting of this research align with international guidelines for AI interventions, including the CONSORT-AI extension [38] and its specific applications in medical imaging [39]. Furthermore, we followed the Checklist for Artificial Intelligence in Medical Imaging (CLAIM) [40], a critical tool whose importance is underscored by its widespread endorsement and the finding that adherence to it is essential for ensuring the quality and reproducibility of AI research in radiology [41]. Ultimately, the entire AI development process was guided by the principles of trustworthy AI, as outlined in the FUTURE-AI consensus recommendations, which prioritize fairness, robustness, and transparency [42].

This study demonstrates that artificial intelligence can substantially improve the reproducibility of breast density (BD) and background parenchymal enhancement (BPE) assessment in contrast-enhanced mammography (CEM). Interobserver variability has long been recognized as a limiting factor in breast imaging [43], with κ values often ranging from fair to moderate for BPE and from moderate to substantial for density classification [44]. Such variability is not trivial: it can directly influence management decisions, including whether women with dense breasts are referred for supplemental imaging or remain in routine screening pathways [45,46,47]. We purposely reported a simple linear baseline (implemented with a spreadsheet tool) to reflect what clinicians might approximate in real-world practice; this baseline performed poorly, highlighting the need for optimized regression or AI architectures.

By introducing computational models, our work reduced this variability, raising agreement from κ values of 0.54 and 0.68 at baseline to 0.82 with AI support, which corresponds to almost perfect concordance. These gains highlight the clinical relevance of algorithmic assistance: when radiologists can rely on reproducible classifications, downstream risk stratification and patient counseling become more consistent.

Our results also emphasize the importance of balancing interpretability and accuracy. While deep neural networks (DNNs) achieved slightly higher R^2^ values than linear regression, Wilcoxon testing confirmed that this difference was not statistically significant (ΔMSE = 0.003, *p* = 0.12). This indicates that, within the constraints of our dataset, linear regression performs comparably to DNNs. Given its transparency and ease of interpretation, linear regression therefore represents a clinically pragmatic solution, whereas DNNs may still be explored in research settings, ideally complemented by explainability tools. This is consistent with prior literature showing that complex models often outperform simpler ones only marginally when datasets are modest in size [48]. More importantly, linear models remain transparent and intuitive: they allow clinicians to understand the contribution of age, density, or enhancement features to the prediction. In breast imaging, where trust and accountability are crucial, this interpretability may outweigh small gains in raw performance. This echoes recommendations from international position papers, which caution against deploying “black box” algorithms without explainability mechanisms [49]. Our findings therefore suggest that linear regression offers a pragmatic, clinically acceptable solution for immediate implementation, while DNNs should be further investigated in research settings, ideally complemented by explainability tools such as SHAP values or saliency maps [50].

The absence of a statistically significant performance difference between linear regression and DNN (*p* = 0.12) suggests that simple, transparent models can achieve results comparable to more complex ‘black box’ architectures in this setting. This has important clinical implications, as interpretability is critical for trust and adoption in breast imaging. The contribution of our work therefore lies not only in demonstrating that AI can standardize BD and BPE in CEM, but also in showing that interpretable models may be sufficient for reliable clinical deployment.

Beyond standardization, our exploratory analysis suggests that CEM may encode information relevant to systemic health. The multi-output model correlated CEM-derived features not only with BD and BPE but also with surrogates for bone mineral density and systolic blood pressure. This finding is conceptually aligned with growing evidence that radiological images can capture systemic biomarkers, reflecting vascular, hormonal, and metabolic states [51,52]. The strong correlation between the bone density surrogate and DXA values (r = 0.82) and the moderate correlation with blood pressure (r = 0.76) support the plausibility of this approach. However, these results should be interpreted cautiously. The analysis was exploratory, not pre-specified, and based on a limited single-center sample. As such, it does not provide clinical validation but rather an initial proof-of-concept for the idea of “multidimensional screening,” in which a single imaging exam might simultaneously provide oncologic, skeletal, and cardiovascular information [53]. Future studies should pursue this concept prospectively, ideally in multicenter settings with larger sample sizes and integrated clinical endpoints.

Our findings also carry practical implications for workflow efficiency. The AI-assisted models reduced false positives in dense breasts by 22% and shortened interpretation time by 35%. These improvements are not only statistically relevant but also operationally meaningful in high-volume screening programs, where radiologists face increasing workload and fatigue [54]. More consistent and faster readings could improve throughput and reduce unnecessary recalls, enhancing patient experience and optimizing use of healthcare resources. Importantly, by reducing subjectivity, AI standardization could also support the creation of large-scale registries with harmonized annotations, enabling more robust population-level studies and benchmarking across institutions.

From a methodological standpoint, the study aligns with emerging standards for AI in medical imaging, including the CLAIM checklist and CONSORT-AI reporting guidelines [55,56]. By providing details on data preprocessing, model architecture, external validation, and performance metrics with confidence intervals, we aimed to maximize transparency and reproducibility. Nevertheless, further steps are needed to reach the level of evidence required for clinical deployment, including uncertainty quantification, fairness assessments across demographic subgroups, and prospective evaluation in randomized screening settings.

The relatively small, single-center dataset inevitably limits the external generalizability of our findings. Although the internal cohort was balanced across BD and BPE categories and external validation on the VinDr-Mammo dataset (*n* = 500) supported reproducibility for density endpoints, contrast-dependent features (BPE) remain unvalidated in a multicenter setting. While cross-modality validation on the VinDr-Mammo dataset (FFDM) confirmed the robustness of density predictions, it cannot validate contrast-dependent features such as BPE. This is an inherent limitation of the current landscape, as large-scale public CEM repositories are not yet available. Consequently, our findings on BPE reproducibility should be regarded as preliminary and hypothesis-generating. Dedicated multicenter CEM datasets will be essential to validate contrast-related endpoints and to establish the full generalizability of the proposed framework.

Moreover, single-center recruitment may reflect local referral patterns, demographic profiles, and imaging protocols, potentially introducing selection bias. Future work should therefore prioritize larger, harmonized, multicenter datasets to confirm reproducibility across diverse populations and technical platforms. Such efforts will also allow integration of clinical, hormonal, and genetic variables, thereby enhancing the robustness and clinical translatability of the framework.

Another limitation is the absence of hormonal and genetic biomarkers, which may influence both BPE and systemic parameters. Finally, while the exploratory multi-output analysis is conceptually attractive, it should be regarded as hypothesis-generating rather than clinically actionable. These limitations underscore the need for larger, multicenter, and prospective studies that integrate imaging with clinical and molecular data to validate the proposed framework.

In summary, this study provides evidence that AI-assisted models can standardize BD and BPE interpretation in CEM, reducing interobserver variability and improving efficiency. Linear regression, in particular, offers a transparent and clinically ready solution, while DNNs and multi-output approaches represent promising avenues for future research. The proof-of-concept integration of systemic indicators, though preliminary, opens the door to reimagining CEM not only as a diagnostic tool for breast cancer but as a platform for multidimensional preventive medicine [57]. The exploratory analysis of systemic surrogates (bone density and blood pressure) should be regarded strictly as proof-of-concept. While the observed correlations are promising, they do not yet constitute evidence of clinical applicability. We position this component as a hypothesis-generating pilot, intended to motivate further multicenter and prospective studies that integrate biological and clinical predictors. By combining rigor in standardization with vision in exploration, this work contributes to the ongoing transformation of breast imaging into a more reproducible, efficient, and integrative discipline.

## 5. Limitations

This study has several limitations that must be acknowledged. First, the sample size was modest (*n* = 213) and derived from a single institution, which inevitably raises concerns regarding the generalizability of the results. Although external validation on the VinDr-Mammo dataset demonstrated stable performance, the generalizability of contrast-dependent features remains uncertain. Only multicenter, prospective studies with harmonized acquisition protocols will establish whether our findings can be extended to broader populations [58]. Second, the analysis relied primarily on imaging-derived features, age, and body mass index. Important biological variables such as menopausal status, hormonal therapy, and genetic markers were not available, even though these factors are known to influence both breast enhancement patterns and systemic health parameters [59]. Their absence limits the comprehensiveness of the predictive models. This limitation is particularly relevant for systemic surrogate predictions, as hormonal and genetic factors strongly modulate both bone density and vascular parameters. Their absence may therefore have attenuated the accuracy of exploratory systemic endpoints.

A further limitation relates to the exploratory multi-output analysis. While the correlations between CEM-derived surrogates and systemic measures such as bone density and blood pressure were encouraging, this arm of the study was not pre-specified, had a smaller effective sample size, and was conducted in a purely retrospective manner. As such, these findings must be considered hypothesis-generating only, not clinically actionable. Prospective validation with ground truth clinical outcomes is essential before any systemic predictions can be integrated into preventive pathways [60].

External validation was cross-modality (CEM-trained model tested on FFDM), which supports robustness for density endpoints but does not validate contrast-dependent features.

Another methodological limitation is that, although confidence intervals were calculated for model performance, uncertainty quantification at the individual patient level was not implemented. Recent consensus papers on trustworthy AI recommend incorporating methods to express prediction uncertainty and to assess fairness across demographic subgroups [61]. This would be particularly important in breast imaging, where misclassification can directly influence patient management and screening recommendations.

## 6. Future Directions

Future studies should systematically incorporate clinical and biological predictors—including menopausal status, hormone replacement therapy, and genetic markers—to improve the comprehensiveness and clinical validity of AI models for both breast-specific and systemic outcomes.

Integration of clinical, hormonal, and genetic biomarkers will help refine model predictions and provide a more complete picture of women’s health. For systemic surrogates, prospective trials should be designed to evaluate the feasibility of using CEM as a gateway to multidimensional preventive screening. These trials should include endpoints such as fracture risk or cardiovascular events to confirm the clinical utility of the multi-output approach.

On the technical side, the development of explainable AI is critical. Incorporating interpretability frameworks, such as feature attribution methods or saliency mapping, will help clinicians understand and trust model outputs. Bayesian or ensemble approaches could also provide patient-level uncertainty estimates, ensuring that predictions are not only accurate but also reliable [62]. Finally, alignment with international reporting standards such as CLAIM and CONSORT-AI should be maintained, ensuring that future studies meet the highest requirements for transparency and reproducibility [63].

In addition, future work should incorporate explainability frameworks, including saliency maps and SHAP values, to visualize the contribution of imaging features and clinical variables to model predictions. Such tools are expected to foster greater clinician trust and to support responsible adoption of AI in breast imaging. Additionally, future studies should benchmark performance against ensemble-based models such as Random Forests and XGBoost. These approaches are well established in biomedical machine learning for their robustness and ability to capture non-linear relationships, and their inclusion will provide a more comprehensive sensitivity analysis of the predictive landscape.

In conclusion, while this study establishes the potential of AI to standardize BD and BPE assessment in CEM and explores the feasibility of multi-output systemic predictions, future work must expand sample size, integrate multimodal data, and adopt explainable and uncertainty-aware models. These steps will be essential to move from proof-of-concept toward clinical translation, transforming breast imaging into a robust, integrative platform for predictive and preventive medicine.

## 7. Conclusions

In this study, we demonstrated that artificial intelligence can significantly improve the reproducibility of breast density and background parenchymal enhancement assessment in contrast-enhanced mammography. By reducing interobserver variability and interpretation time while maintaining transparency through interpretable linear models, AI provides an immediately applicable tool to support clinical practice. At the same time, our exploratory multi-output framework suggests that CEM may carry information relevant not only to oncologic risk but also to systemic health, including bone and cardiovascular parameters. These findings highlight the dual contribution of AI: on the one hand, offering a practical solution for standardization, and, on the other, opening a conceptual pathway toward multidimensional preventive medicine. While further multicenter, prospective, and uncertainty-aware studies are needed before translation into routine care, this work underscores the potential of CEM to evolve from a diagnostic exam into an integrated platform for predicting women’s health [64].

## Figures and Tables

**Table 1:** Baseline characteristics of the study cohort.

Section	Measure	Value
Age Statistics	Mean ± SD	58.3 ± 11.2
Age Statistics	Interquartile Range	51–67
Breast Density Distribution	A	12%
	B	29%
	C	38%
	D	21%
BPE Distribution	Minimal	55%
	Mild	30%
	Moderate	12%
	Marked	3%

Baseline characteristics of the study population (*n* = 213). The table reports mean and interquartile range of patient age, breast density (BD) distribution according to BI-RADS categories A–D, and background parenchymal enhancement (BPE) distribution (minimal–marked). BD = breast density; BPE = background parenchymal enhancement; BI-RADS = Breast Imaging Reporting and Data System.

**Table 2:** Model performance on the internal dataset.

Model	MSE	R^2^	AUC	Precision	Recall
Simple linear baseline	0.864 (95% CI: 0.822–0.874)	0.144 (95% CI: 0.130–0.151)	–	–	–
Linear Regression (scikit-learn)	0.641 (95% CI: 0.543–0.747)	0.203 (95% CI: 0.199–0.241)	0.73 (95% CI: 0.671–0.789)	0.70 (95% CI: 0.634–0.77)	0.68 (95% CI: 0.57–0.70)
Deep Neural Network (DNN)	0.638 (95% CI: 0.540–0.741)	0.233 (95% CI: 0.124–0.339)	0.75 (95% CI: 0.694–0.809)	0.72 (95% CI: 0.649–0.792)	0.69 (95% CI: 0.615–0.762)

Performance of simple linear baseline, linear regression, and deep neural network (DNN) models on the internal dataset. Results are reported as mean squared error (MSE), explained variance (R^2^), and, where applicable, classification metrics (area under the curve [AUC], precision, recall). MSE = mean squared error; R^2^ = coefficient of determination; AUC = area under the curve; DNN = deep neural network.

**Table 3:** External validation (VinDr-Mammo, *n* = 500 BI-RADS C/D).

Model	MSE	AUC	Notes
Linear Regression (scikit-learn)	0.652	0.74	Performance stable on BI-RADS C/D subset
Deep Neural Network (DNN)	0.652	0.75	Performance stable on BI-RADS C/D subset

External validation on the VinDr-Mammo dataset (*n* = 500, BI-RADS C/D). Both linear regression and DNN models demonstrated stable generalization with consistent MSE and AUC values. Notes indicate performance stability across higher-density subgroups. MSE = mean squared error; AUC = area under the curve; DNN = deep neural network; BI-RADS = Breast Imaging Reporting and Data System.

**Table 4:** Sensitivity analysis excluding cases with marked BPE (3% of the cohort, n ≈ 6).

Outcome	Full Cohort	Excluding Marked BPE
κ (BPE)	+0.28	+0.28
Reading time	−35% (6.3→4.1 min)	−35% (6.3→4.1 min)
MSE	−0.223 (−25.8%)	−0.222 (−25.7%)

Effect sizes for κ gains, reading-time reduction, and MSE reduction were essentially unchanged, confirming that overall results are not driven by extreme BPE cases.

**Table 5:** Calibration metrics for AI models.

Model	Calibration Slope (95% CI)	Calibration Intercept (95% CI)
Linear regression	0.96 (0.88–1.04)	+0.02 (−0.03 ± 0.07)
Deep neural network	1.01 (0.93–1.09)	−0.01 (−0.05 ± 0.04)

Ideal values are slope = 1 and intercept = 0. Both models demonstrated near-ideal calibration, indicating that predicted probabilities are well aligned with observed frequencies.

## Data Availability

The data presented in this study are available upon request from the corresponding author.
